# Liver transplantation in a child with liver cirrhosis caused by langerhans cell histiocytosis: a case report

**DOI:** 10.1186/s12887-021-03090-4

**Published:** 2022-01-03

**Authors:** Qi Wang, Shuguang Jin, Bo Xiang, Jing Chen

**Affiliations:** Department of Pediatric Surgery, West China Hospital, Sichuan University, Chengdu, 610041 China

**Keywords:** Langerhans cell histiocytosis, Sclerosing cholangitis, Liver cirrhosis, Liver transplantation, Case report

## Abstract

**Background:**

Langerhans cell histiocytosis (LCH) is a rare condition that has a variety of clinical manifestations. But LCH in children localized only in the hepatobiliary system is unusual.

Case presentation.

Here we reported a rare case of a 2-year-old boy who was serendipitously found to have elevated liver enzymes while undergoing treatment of a perianal abscess. After a period of earlier conservative treatment in another hospital, the perianal abscess had resolved but the levels of liver enzymes were still rising slowly. The child was then referred to our institution for a definitive diagnosis. After laboratory tests, imaging and pathological examinations, a diagnosis of liver cirrhosis and sclerosing cholangitis was established, although the cause was unclear. Subsequently, living-donor liver transplantation was performed due to deterioration in liver function. Following successful liver transplantation, a diagnosis of LCH localized only within the hepatobiliary system was finally confirmed, based on additional pathological and imaging investigation. Additionally, the *BRAF V600E* mutation in this patient was also confirmed. The child has now recovered without evidence of LCH recurrence.

**Conclusions:**

LCH localized only within the hepatobiliary system is unusual. The presence of unexplainable sclerosing cholangitis and liver cirrhosis in any child should raise the suspicion of LCH.

**Supplementary Information:**

The online version contains supplementary material available at 10.1186/s12887-021-03090-4.

## Background

Langerhans cell histiocytosis (LCH) is a rare condition characterized by the accumulation of CD1a or Langerin-positive dendritic cells in various organs, often leading to organ dysfunction [1]. Therefore, the clinical maifestations of LCH are highly variable, ranging from a self-limiting disease to fatal multi-organ involvement [2]. The most common affected organs are the bone (80%), the skin (33%), and the pituitary gland (25%) [1]. When it comes to liver, LCH may cause sclerosing cholangitis, biliary cirrhosis and organ damage requiring liver transplantation[3]. However, only a limited number of articles have reported on primary LCH localized to the hepatobiliary system in pediatric patients [4, 5].

Herein, we reported the clinical details of a rare pediatric case who underwent liver transplantation due to liver cirrhosis caused by LCH restricted to the hepatobiliary system.

### Case presentation

A 2-year-old boy was referred to our hospital for the definitive diagnostic workup due to elevated liver enzymes. Approximately 5 months earlier during treatment for a perianal abscess, the child was found to have an abnormal high level of serum aspartate aminotransferase (AST) and alanine aminotransferase (ALT). After resolution of the perianal abscess, the liver enzyme levels continued to slowly rise, although symptoms such as fatigue, anorexia, jaundice or pruritus were not apparent during this period. The child was treated at the initial hospital with magnesium isoglycyrrhizinate and ademetionine 1,4-butanedisulfonate, aiming to improve liver function, but the effects were limited. The child was otherwise in good health with no family or genetic history of liver dysfunction, and no other family member has the similar clinical condition.

**Fig. 1:**
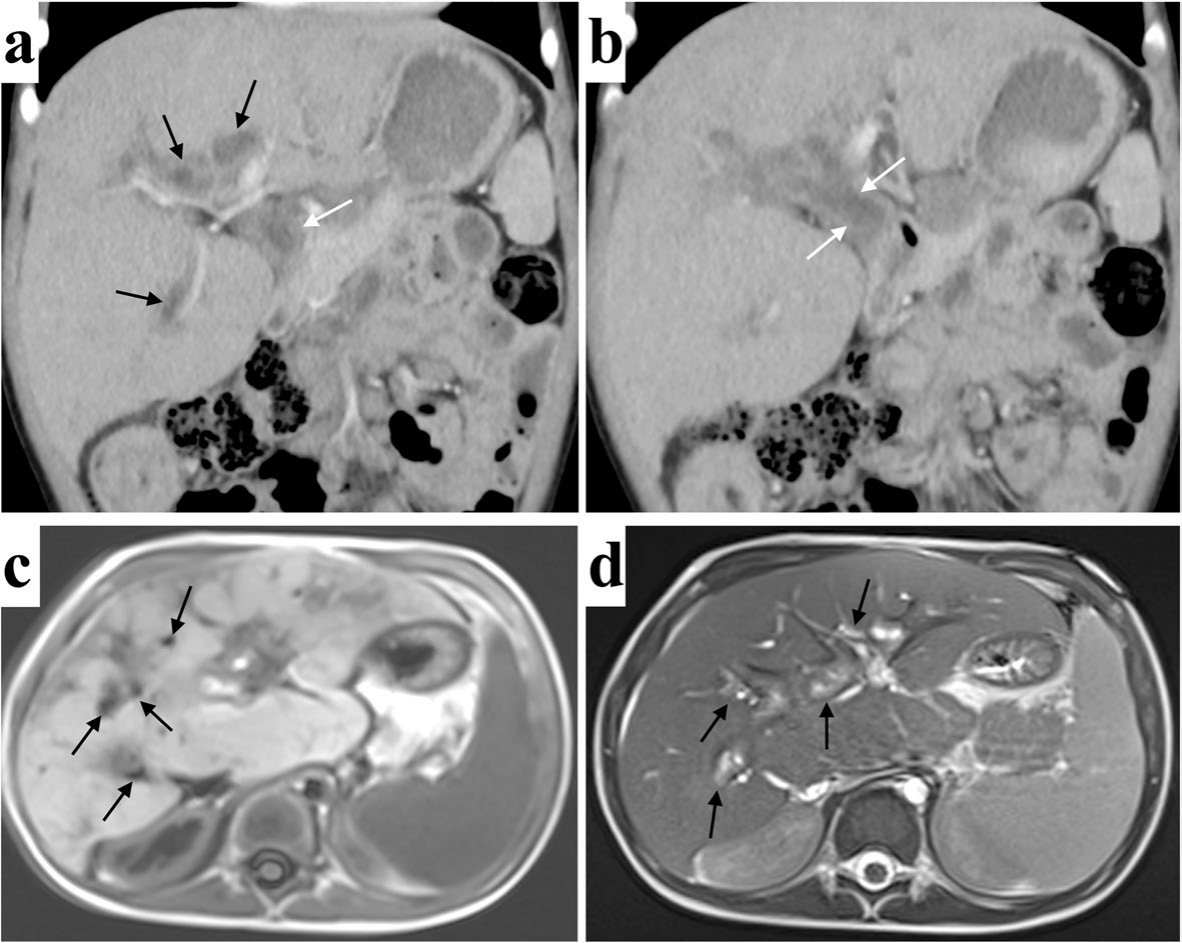
Contrast-enhanced computed tomography (CT) (coronal view) and Magnetic resonance imaging (MRI) of the patient’s abdomen. **a** The dilated intrahepatic bile duct (black arrows) and extrahepatic bile duct (white arrows) in CT. **b** The dilated extraheaptic bile duct (white arrows) in CT. **c** The dilated intrahepatic bile duct (black arrows) in MRI T1-weighted image. **d** The dilated intraheaptic bile duct (black arrows) in MRI T2-weighted image

After a detailed physical examination, the child was found to be well developed with no pathological signs except a palpable liver approximately 4 cm below the right costal margin. Laboratory data demonstrated elevation of liver enzymes (ALT 225 U/L, AST 261 U/L, total bilirubin 38.7 umol/L, direct bilirubin 34.1 umol/L, albumin 43.1 g/L, alkaline phosphatase 1462 U/L, glutamyl transpeptidase 744 U/L, total bile acid 156.2 umol/L). Computed tomography (CT) and magnetic resonance imaging (MRI) scans indicated altered liver morphology, including atrophy of the left hepatic lobe, enlargement of the right hepatic lobe and caudate lobes, irregular dilation of the intrahepatic and extrahepatic bile ducts, multiple choleliths in the bile duct system and thickening of the bile duct wall (Fig. 1). These findings pointed to liver cirrhosis and sclerosing cholangitis. To further clarify the diagnosis, an ultrasound-guided liver biopsy was performed. Pathological findings revealed bile stasis, damage and regeneration of liver cells and the small bile duct, proliferation of fibrous tissue, dilation of the portal area, and infiltration of inflammatory cells (Fig. S1). A diagnosis of liver cirrhosis and sclerosing cholangitis was therefore confirmed, although the etiology of the lesion was unclear.

Meanwhile, the liver cirrhosis was progressive, the patient’s Child–Pugh classification was deteriorated from grade A to grade B in approximately 3 weeks (Fig. S2). We concluded that this patient would soon require a liver transplantation. Living-donor liver transplantation from his mother was performed about 1 month after the liver biopsy in our hospital. During transplantation surgery, the entire liver and extrahepatic bile duct of the recipient was removed, and cholangiojejunostomy Roux-en-Y was performed.

**Fig. 2:**
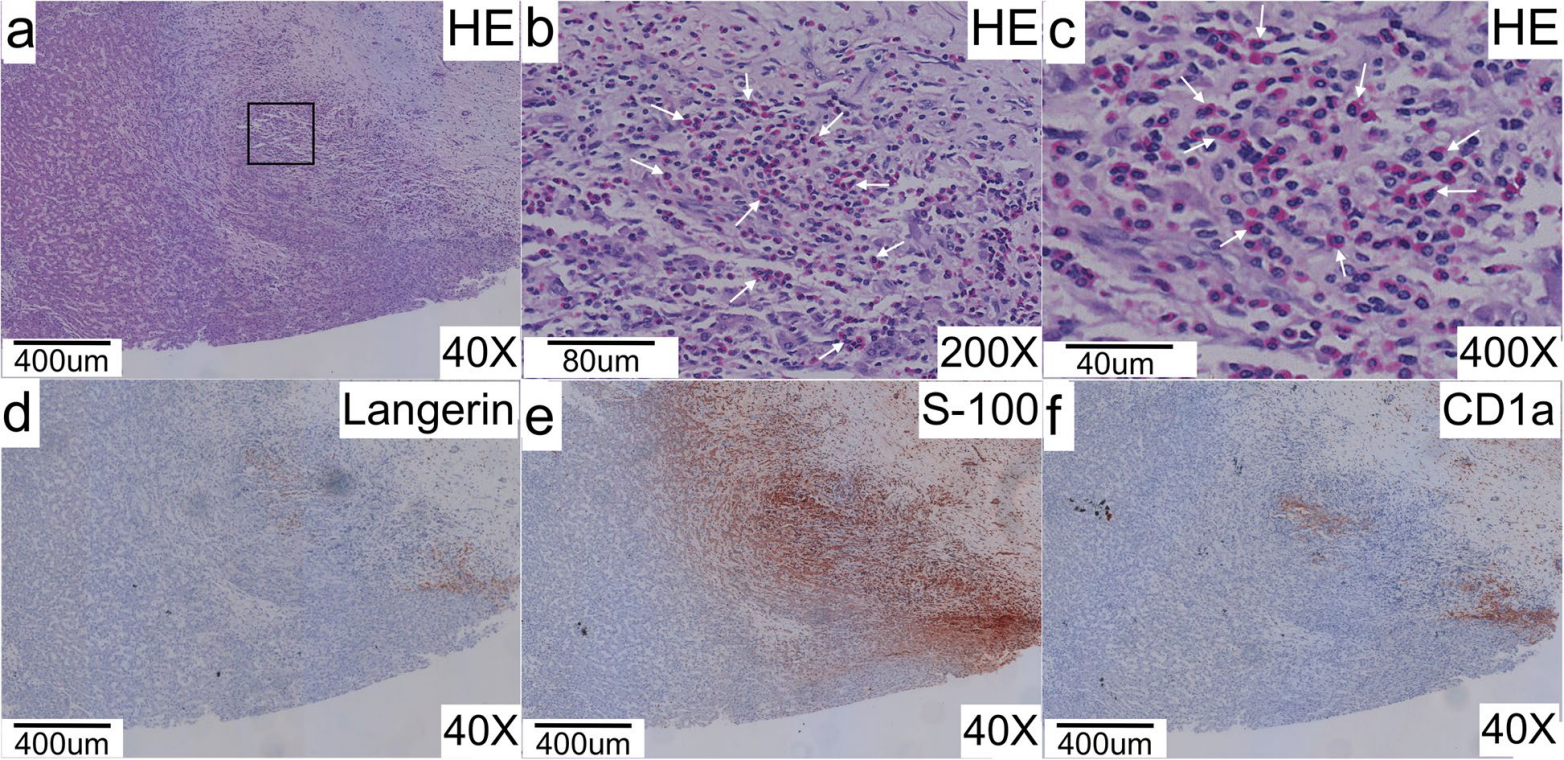
Pathological findings and immunohistochemical staining of the recipient’s liver. **a** Hematoxylin and eosin staining of the recipient’s liver. **b,c** Enlarged view of liver tissue in the black box in **a,** with diffuse infiltration of eosinophils cells characterized by grooved and convoluted nuclei with a scattered chromatin texture (white arrows). **d** Immunohistochemical staining for Langerin. **e** Immunohistochemical staining for S-100 protein. **f** Immunohistochemical staining for CD1a

**Fig. 3:**
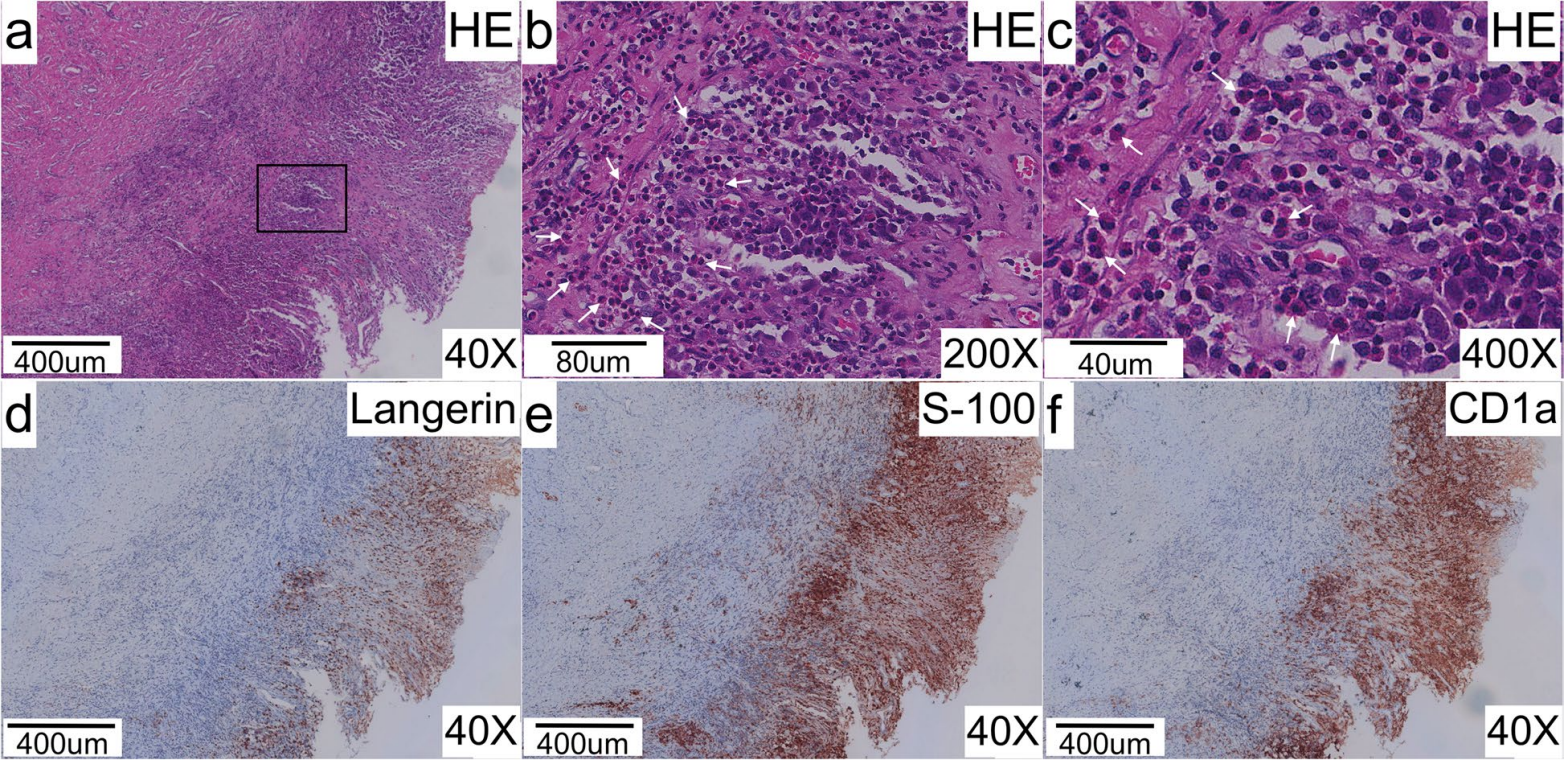
Pathological findings and Immunohistochemical staining of the recipient’s extrahepatic bile duct. **a** Hematoxylin and eosin staining of the recipient’s extrahepatic bile duct. **b,c** Enlarged view of duct tissue within the black box in **a,** with diffuse infiltration by eosinophils cells characterized by grooved and convoluted nuclei with scattered chromatin texture (white arrows). **d** Immunohistochemical staining for Langerin. **e** Immunohistochemical staining for S-100 protein. **f** Immunohistochemical staining for CD1a

The macroscopic appearance of the recipient’s liver was consistent with the conclusions of preoperative imaging examination (Fig. S3). Microscopically, in addition to the pathological findings of the liver cirrhosis and sclerosing cholangitis described above, diffuse infiltration of eosinophils cells characterized by grooved and convoluted nuclei with scattered chromatin structure were observed in the liver (Figs. 2a, b, c) and extrahepatic bile duct (Figs. 3a, b, c). Furthermore, immunohistochemical staining demonstrated that these cells were positive for Langerin (Figs. 2d & 3d), S-100 protein (Figs. 2e & 3e), and CD1a (Figs. 2f & 3f), consistent with the characteristics of Langerhans cells. We confirmed a diagnosis of LCH, although the extent of the disease remained unclear. Following whole-body scanning, high-resolution CT scanning of the lung, and MRI examination of the head, no other lesions were found. Ultimately, a diagnosis of LCH, localized only in the hepatobiliary system, was established. Additionally, the *BRAF V600E* mutation was also confirmed for this patient after genetic analysis by the pathology department in our hospital.

The child and mother recovered well after living-donor liver transplantation, and were discharged on postoperative day 21. Tacrolimus was used as an immunosuppressor. Due to the presence of the *BRAF V600E* mutation, dabrafenib was prescribed for the treatment of LCH after fully communicating with the child’s parent and obtaining consent. Now, the child is well with normal liver function with no significant postoperative complication and no evidence of recurrence of LCH at the 22 months follow-up (ALT 17 U/L, AST 34 U/L, total bilirubin 6.9 umol/L, direct bilirubin 3.5 umol/L, alkaline phosphatase 299 U/L, glutamyl transpeptidase 33 U/L, total bile acid 13.5 umol/L) (Fig. S2; Fig. S4).

## Discussion and Conclusions

LCH generally occurs in children ≤ 4 years old at an incidence of 2.9 cases per million, considered extremely rare in adults with an incidence of 1–2 cases per million[2, 6, 7]. Histological examination with immunophenotyping was required to make the diagnosis of LCH. Identification of characteristic cells and confirmation of the presence of markers such as CD1a and/or Langerin (CD207) are definitive [3].

Because Langerhans cells can accumulate in almost any tissue or organ, the clinical presentation of LCH highly variable, from isolated, self-healing skin and bone lesions to life-threatening multi-organ involvement[3]. This characteristic of LCH increases the difficulty of diagnosis, or the risk of misdiagnosis or delayed diagnosis. In this case, the patient was almost totally asymptomatic with abnormal liver function detected accidentally. A diagnosis of LCH was not confirmed for almost 7 months. During this period, the patient’s liver function deteriorated rapidly, so the child had to underwent a liver transplant. However, a diagnosis of LCH was not considered from the biopsy obtained from the initial surgery. We feel, on reflection, that the outcome from such a diagnosis may be better if a definitive diagnosis could be made as early as possible. Therefore, in biopsies that demonstrate periportal lymphocytic infiltration, bile duct inflammation, and loss of bile ducts from any patient with unexplained liver cirrhosis and sclerosing cholangitis, LCH should be considered. Although LCH is considered as a non-hereditary disease, it has been reported to affect other family members [8]. So the detailed investigation about other family members is quite necessary. In addition, previous studies reported that 75% of children with LCH and sclerosing cholangitis do not respond to chemotherapy, all this part of patients required liver transplantation[3, 9].

Depending on the extent of involvement, LCH is currently classified as three distinct forms: single-system single site (SS-s), single-system multi-site (SS-m) and multisystem disease (MS)[3, 10–12]. The ratio of these three forms was reported to be almost 1:1:1 in a Japanese study[13]. Although LCH has shown an excellent 2-year overall survival of 98.7% and the prognosis for single system LCH is generally good [2, 13], liver involvement can dramatically change a patient’s prognosis and treatment, with the 5-year overall survival rate was reported to be only 25% in this subgroup of patients[14–16]. Treatment for LCH depends on the form of disease, and can range from observation to surgical intervention, radiotherapy, or chemotherapy[3]. Unfortunately, due to the limited number of cases[4, 5], optimal treatment regimens for LCH localized only in the hepatobiliary system remains unclear. Nevertheless, reported cases of living-donor liver transplantation in LCH related sclerosing cholangitis and liver cirrhosis have all been successful with an overall survival rate of approximately 87% after a mean follow-up of 3.4 years[4, 17]. So far, with the detailed and comprehensive radiological, pathological and clinical data throughout the whole process, the outcome of this case is also considered successful.

Furthermore, the *BRAF V600E* mutation, which indicates a high risk of recurrence and a high permanent complication rate[18, 19], was also detected in our patient. The mutation may also contribute to the pathogenesis of LCH through activation of the MAPKinase RAS-RAF-MEK-ERK cell signaling pathway, and has been identified in more than half of the childhood LCH[3, 19, 20]. Therefore, MAPK pathway inhibitors (including dabrafenib and vemurafenib) have been applied as targeted therapy in LCH patients recently [21–24]. Similar with the patients who were treated with chemotherapy, the recurrence can also occur in LCH patients treated with targeted therapy, and the rate was reported to be as high as 84% after 12 months of the discontinuation in refractory or relapse LCH[21]. However, targeted therapy can achieve a response rate about 86% in LCH patients during its application with more tolerable adverse effects, especially in patients who have had little or no response to previous treatments and suffering refractory or relapse LCH [21–24]. Thus, targeted therapy (dabrafenib) was used in this patient after careful evaluation. Moreover, immunosuppressive agents have also been reported to assist in preventing recurrence[4]. In this case, the child is developing uneventfully with no evidence of recurrence by now.

Finally, we made a conclusion that LCH localized only in the hepatobiliary system is rare, and the presence of unexplainable sclerosing cholangitis and liver cirrhosis in any child should raise suspicion of LCH.

## Supplementary Information

**Additional file 1:** **Figure S1.** Hematoxylin and eosin staining of liver biopsy.① the damage and regeneration of liver cells and pseudolobule formation. ② dilation of portal area, proliferation of fibrous tissue and small bile duct. ③ infiltration of inflammation cells. **Figure S2.** The changes of liver function in this LCH patient. The patient was found to have an abnormal liver function at 20 months old, and was referred to our hospital at 25 months old. Then the living-donor liver transplantation was perfomed when he was 27 months old. ALT: alanine aminotransferase; AST: aspartate aminotransferase; PT: prothrombin time; TB: total bilirubin; ALB: albumin. **Figure S3.** Macroscopic appearance of the recipient’s liver. a The diaphragmatic surface of liver. b The visceral surface of liver. **Figure S4.** The enhanced CT of the chest and ultrasound examination of the liver. a-b: the enhanced CT of the chest was performed when the patient was 39 months old and the results were normal; c-d: the ultrasound examination of the liver was performed regularly every 3-6 month since the liver transplantation, and the results were all shown that the echo of the transplanted liver parenchyma was uniform and no dilation of the intrahepatic biliary tract was observed, the presented images were performed when the patient was 45 months old.

## Data Availability

The data used during the current study are available from the corresponding author on reasonable request.

## Abbreviations

LCH: Langerhans cell histiocytosis
AST: Aspartate aminiotrasferase
ALT: Alanine aminotrasferase
CT: Computed tomography
MIRI: Magnetic resonance imaging
