# Supplementary material for: Liver transplantation in a child with liver cirrhosis caused by langerhans cell histiocytosis: a case report
Source: BMC Pediatr. 2022 Jan 3;22:18. doi: 10.1186/s12887-021-03090-4 (PMC8721976; doi:10.1186/s12887-021-03090-4)
Supplement: Supplementary file 1 — Additional file 1: Figure S1. Hematoxylin and eosin staining of liver biopsy.① the damage and regeneration of liver cells and pseudolobule formation. ② dilation of portal area, proliferation of fibrous tissue and small bile duct. ③ infiltration of inflammation cells. Figure S2. The changes of liver function in this LCH patient. The patient was found to have an abnormal liver function at 20 months old, and was referred to our hospital at 25 months old. Then the living-donor liver transplantation was perfomed when he was 27 months old. ALT: alanine aminotransferase; AST: aspartate aminotransferase; PT: prothrombin time; TB: total bilirubin; ALB: albumin. Figure S3. Macroscopic appearance of the recipient’s liver. a The diaphragmatic surface of liver. b The visceral surface of liver. Figure S4. The enhanced CT of the chest and ultrasound examination of the liver. a-b: the enhanced CT of the chest was performed when the patient was 39 months old and the results were normal; c-d: the ultrasound examination of the liver was performed regularly every 3-6 month since the liver transplantation, and the results were all shown that the echo of the transplanted liver parenchyma was uniform and no dilation of the intrahepatic biliary tract was observed, the presented images were performed when the patient was 45 months old. [file 12887_2021_3090_MOESM1_ESM.pdf]

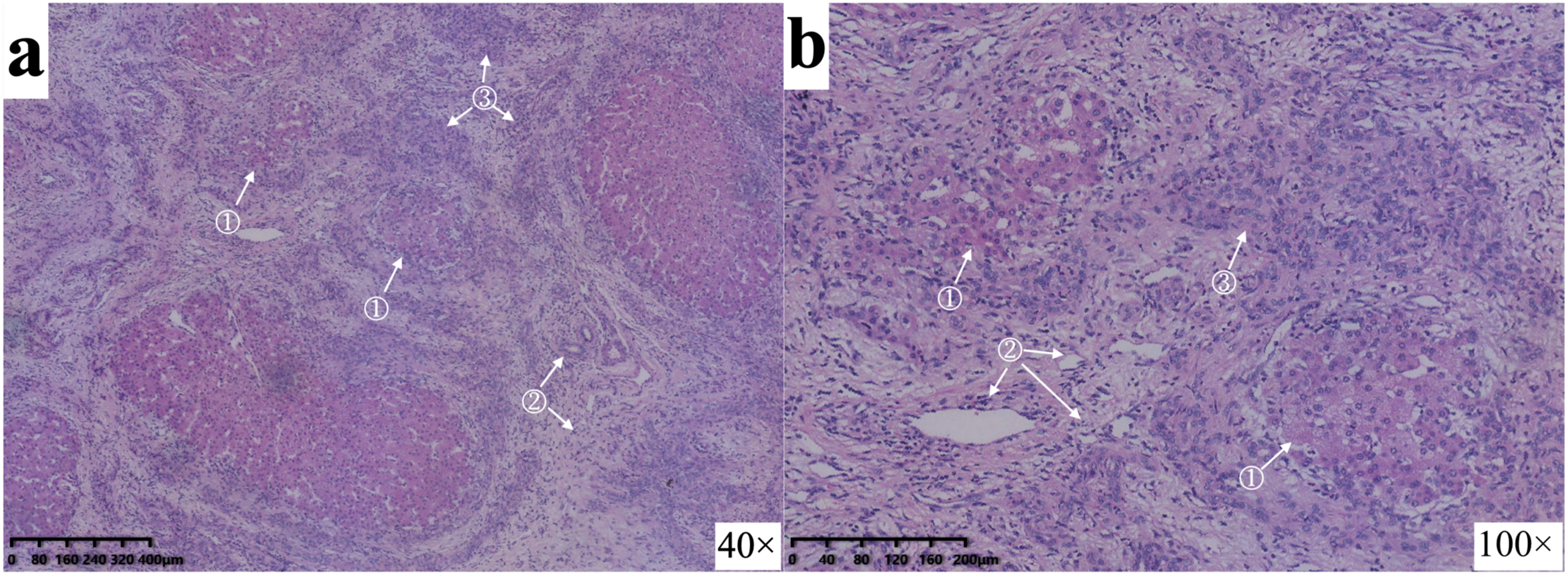

Figure S1. Hematoxylin and eosin staining of liver biopsy.① the damage and regeneration of liver cells and pseudolobule formation. ② dilation of portal area, proliferation of fibrous tissue and small bile duct. ③ infiltration of inflammation cells.

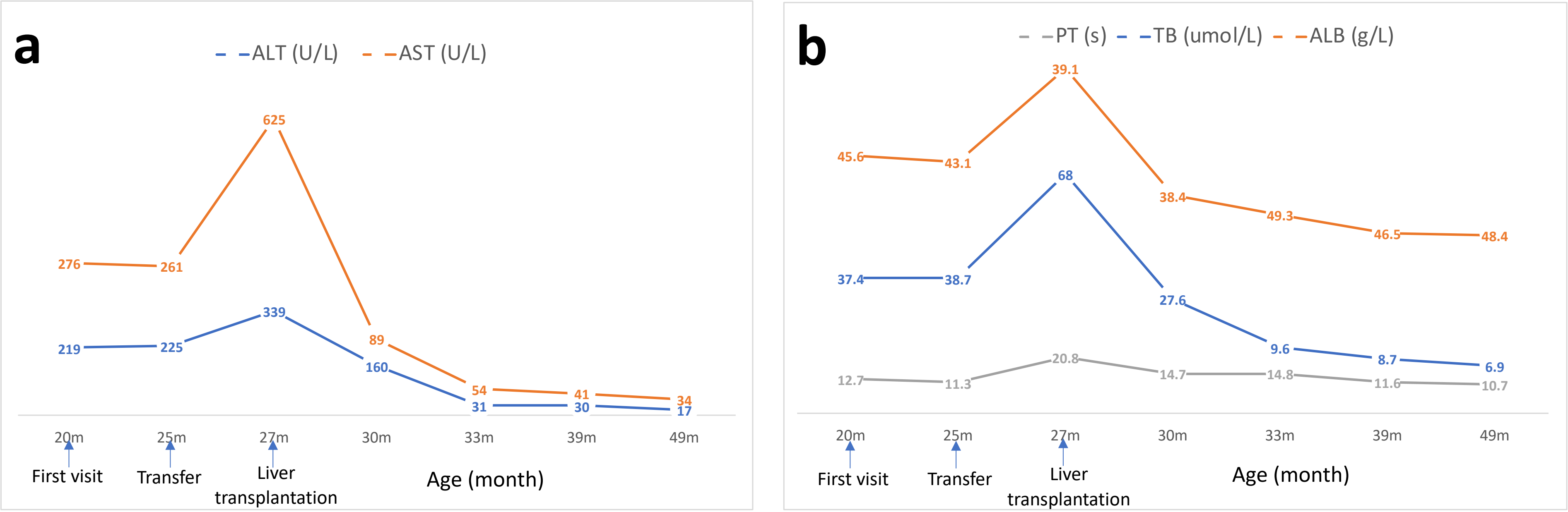

Figure S2. The changes of liver function in this LCH patient. The patient was found to have an abnormal liver function at 20 months old, and was referred to our hospital at 25 months old. Then the living-donor liver transplantation was perfomed when he was 27 months old.
ALT: alanine aminotransferase; AST: aspartate aminotransferase; PT: prothrombin time; TB: total bilirubin; ALB: albumin.

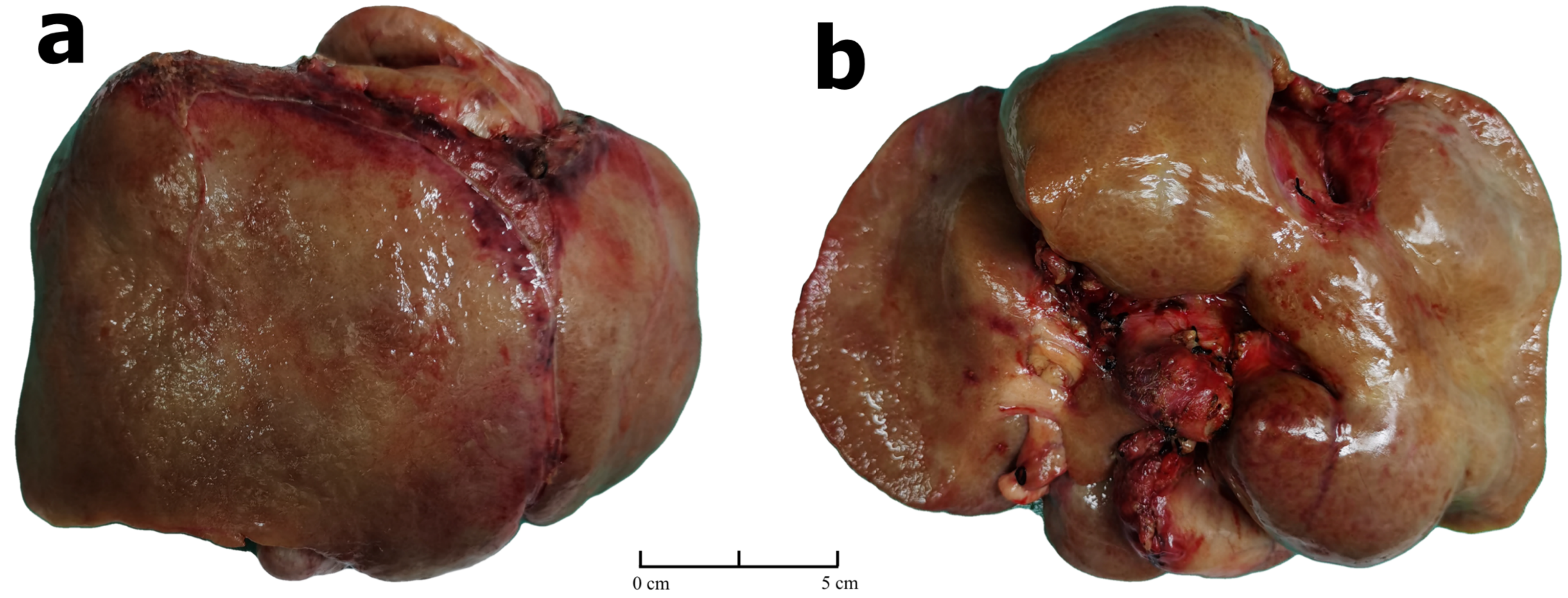

Figure S3. Macroscopic appearance of the recipient's liver. **a** The diaphragmatic surface of liver. **b** The visceral surface of liver.

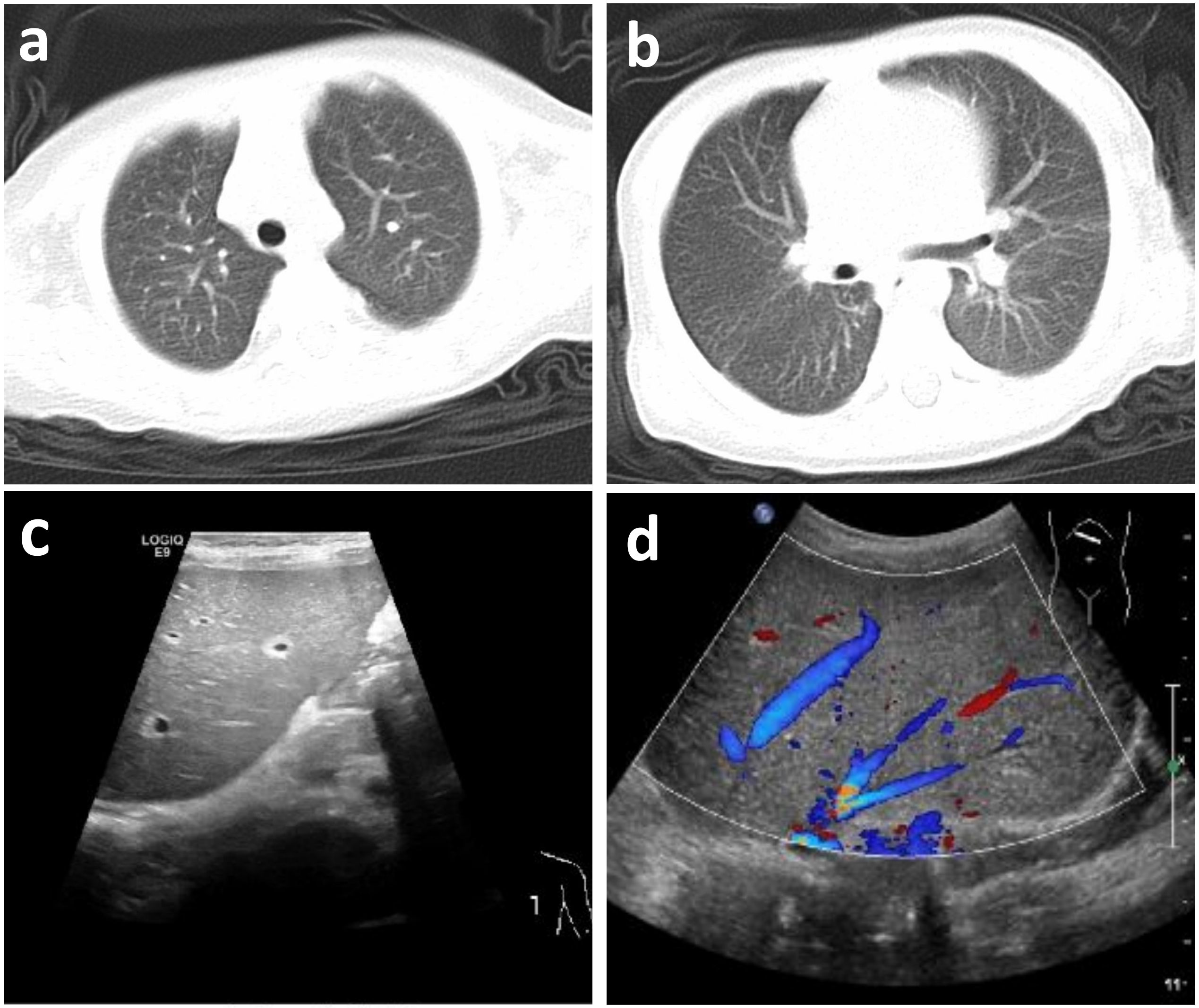

Figure S4. The enhanced CT of the chest and ultrasound examination of the liver.
a-b: the enhanced CT of the chest was performed when the patient was 39 months old and the results were normal;
c-d: the ultrasound examination of the liver was performed regularly every 3-6 month since the liver transplantation, and the results were all shown that the echo of the transplanted liver parenchyma was uniform and no dilation of the intrahepatic biliary tract was observed, the presented images were performed when the patient was 45 months old.
